# MicroRNA-494 induces breast cancer cell apoptosis and reduces cell viability by inhibition of nicotinamide phosphoribosyltransferase expression and activity

**DOI:** 10.17179/excli2018-1748

**Published:** 2019-09-12

**Authors:** Seyedeh Sara Ghorbanhosseini, Mitra Nourbakhsh, Mohammad Zangooei, Zohreh Abdolvahabi, Zahra Bolandghamtpour, Zahra Hesari, Zeynab Yousefi, Ghodratollah Panahi, Reza Meshkani

**Affiliations:** 1 Department of Biochemistry, Faculty of Medicine, Iran University of Medical Sciences, Tehran, Iran; 2 Department of Biochemistry, Faculty of Medicine, Birjand University of Medical Sciences, Birjand, Iran; 3 Department of Biochemistry and Genetics, Cellular and Molecular Research Center, Qazvin University of Medical Sciences, Qazvin, Iran; 4 Department of Molecular Medicine, Faculty of Advanced Technologies in Medicine, IUMS, Tehran, Iran; 5 Laboratory Sciences Research Center, Golestan University of Medical Sciences, Gorgan, Iran; 6 Department of Laboratory Sciences, Faculty of Paramedicine, Golestan University of Medical Sciences, Gorgan, Iran; 7 Department of Biochemistry, Faculty of Medicine, Tehran University of Medical Sciences, Tehran, Iran

**Keywords:** breast cancer, NAMPT, apoptosis, miR-494, microRNA

## Abstract

Breast cancer (BC) is the most prevalent cause of cancer-related death in women worldwide. BC is frequently associated with elevated levels of nicotinamide phosphoribosyltransferase (NAMPT) in blood and tumor tissue. MicroRNA-494 (miR-494) has been described to play key anti-tumor roles in human cancers. The aim of the present study was to investigate the inhibitory effect of miR-494 on NAMPT-mediated viability of BC cells. In this experimental study, MCF-7 and MDA-MB-231 cells were cultured and then transfected with miR-494 mimic, miR-494 inhibitor and their negative controls. The mRNA and protein expression of NAMPT were assessed using real-time PCR and Western blotting, respectively. Subsequently, intracellular NAD levels were determined by a colorimetric method. Finally, cell apoptosis was examined by flow cytometry. Bioinformatics evaluations predicted NAMPT as a miR-494 target gene which was confirmed by luciferase reporter assay. Our results showed an inverse relationship between the expression of miR-494 and NAMPT in both MCF-7 and MDA-MB-231 cell lines. miR-494 significantly down-regulated NAMPT mRNA and protein expression and was also able to reduce the cellular NAD content. Cell viability was decreased following miR-494 up-regulation. In addition, apoptosis was induced in MCF-7 and MDA-MB-231 cells by miR-494 mimic. Our findings indicate that miR-494 acts as a tumor suppressor and has an important effect in suppressing the growth of BC cells through NAMPT. Therefore, miR-494 might be considered as a novel therapeutic target for the management of human breast cancer.

## Introduction

Breast cancer (BC) is the most prevalent malignancy in women worldwide (Torre et al., 2017[42], Ferlay et al., 2010[14]). This disease has high occurrence and mortality rate in many developing countries (Özmen, 2011[31]). Its rate is also growing strongly in South America, Africa and Asia (Ghoncheh et al., 2016[17], Bhikoo et al., 2011[5]). 

Breast cancer is accompanied by many molecular and biochemical alterations. One of the changes observed in patients suffering from BC is the elevated levels of nicotinamide phosphoribosyltransferase (NAMPT) (also known as PBEF or visfatin) in their blood and tumor tissue (Lee et al., 2011[26], Cymbaluk-Płoska et al., 2018[11]). NAMPT, a rate-limiting enzyme in mammalian salvage pathway of nicotinamide adenine dinucleotide (NAD) synthesis, catalyzes the condensation of nicotinamide with 5-phosphoribosyl-1-pyrophosphate (PRPP) to produce nicotinamide mononucleotide (NMN). Human NAMPT consists of 491 amino acids with a molecular weight of 52 kDa (Burgos, 2011[8]; Martin et al., 2001[28]). A number of cancers show increased expression of NAMPT (Dalamaga, 2012[12]; Wang et al., 2011[46]; Bi et al., 2011[7]). Therefore, NAMPT could be a useful biomarker for tumorigenesis or for the prediction of cancer survival (Wu et al., 2012[48]; Bi and Che, 2010[6]). 

Over the past decades, researchers' attention has focused mostly on the involvement of deregulated genes at the levels of DNA, protein, RNA and microRNAs (miRNAs or miRs). MicroRNAs are small non-coding RNA molecules of about 22 nucleotides which regulate gene expression through base-pairing with target mRNAs, resulting in translational suppression or mRNA cleavage (Peng et al., 2016[32]; Khoshnaw et al., 2009[25]). Primary *in silico* analyses have shown that up to 92 % of human genes can be controlled by miRNAs (Wang et al., 2010[46]; Zadeh et al., 2015[52]). An interference technology by miRNA has become a research tool for genetic studies and a new class of drugs has been designed to silence disease-causing genes, although it is still in the development stage. miRNAs are involved in several cellular procedures in BC cells (Wang and Luo, 2015[45]). 

miR-494 seems to play diverse roles in different cancer types. However, miR-494 is down-regulated in several kinds of human cancers including BC, and its augmentation leads to inhibition of cancer progression. Therefore miR-494 is suggested to act as a novel tumor suppressor (Cheng et al., 2018[10]; Olaru et al., 2011[30]).

Our preliminary bioinformatics evaluation showed that the 3'-UTR of the NAMPT mRNA is a target for miR-494. However, their interactions have not been studied experimentally. Thus, the aim of the present study was to investigate the effect of miR-494 on NAMPT and subsequently the cell viability. 

## Materials and Methods

### Cell culture

The human BC cell lines MDA-MB-231 and MCF-7 as well as HEK-293 T cells were obtained from the Cell Bank of the Iranian Biological Resource Center (Tehran, Iran). The cells were grown in DMEM/F12 medium supplemented with 10 % fetal bovine serum (FBS), penicillin (100 U/mL), and streptomycin (100 μg/mL). For MCF-10A (as non-tumorigenic human breast) cells, DMEM-F12 medium was supplemented with 5 % horse serum, 20 ng/ml epidermal growth factor (EGF), 10 μg/ml insulin, 100 ng/ml cholera toxin and 0.5 μg/ml hydrocortisone. All cell lines were kept at 37 °C in 5 % CO_2_ and 95 % humidified air atmosphere.

### Cell transfection 

Cells were seeded in 6-, 12- or 96-well plates 24 h before transfection. Transfection was performed by miR-494 mimic, miR-494 inhibitor or their negative controls using Lipofectamine 2000 (Invitrogen, Carlsbad, CA, USA). After 5 h of transfection, Opti-MEM medium was removed and fresh growth medium was added to each well and the plate was incubated at 37 °C with 5 % CO_2_. After 48 h of transfection, cells were harvested for the subsequent experiments. To confirm the transfection, the FAM-labeled miRNA was transfected into the cells and observed by fluorescence microscope 8-24 h after transfection.

### Target prediction 

The targeting and alignment of miR-494 with the 3'-UTR of NAMPT as well as the binding strengths and scores were checked with miRNA target prediction sites, including microRNA.org (miRaNda algorithm) (Betel et al., 2008[4]), MiRmap (Vejnar and Zdobnov, 2012[43]) and TargetScan (Agarwal et al., 2015[2]).

### Western blotting

After transfection with miRNA, cells were harvested and washed with cold phosphate buffered saline (PBS), and then subjected to lysis by RIPA buffer [Tris-HCl pH 8.0, NaCl, NP-40, Sodium deoxycholate, SDS, Na_3_VO_4_, EDTA] containing the complete protease and phosphatase inhibitor cocktail (Sigma-Aldrich, Germany). The protein concentration was determined using a micro-BCA protein assay kit (Thermo Fisher Scientific, USA). Cell lysate (40 μg total protein) were separated on SDS-PAGE gel and transferred to polyvinylidene difluoride PVDF membranes (Roche Applied Science, Germany). The membranes were first blocked with 5 % skim milk/Tris-buffered saline containing 0.1 % Tween-20 (TBST) at room temperature (RT) for 30 minutes, after which the membranes were incubated overnight at 4 °C. Membranes were incubated with Primary antibodies against NAMPT (1;1000), and GAPDH (1:3000) overnight at 4 °C followed by incubation with HRP-conjugated anti-rabbit secondary antibody (1:10,000 dilution) for 1 h at RT. The membranes were then washed 3-6 times with TBST and the bands were visualized using an ECL Western blotting detection system (GE Amersham, London, UK). ImageJ software (NIH, Bethesda, MD, USA) was used for the densitometric evaluation of protein bands. The results were normalized to the GAPDH band intensity as internal control.

### Total RNA isolation and quantitative real-time PCR (qRT-PCR)

Total RNA from cell lines was extracted with miRCURY™ RNA Isolation Kit-Cell & Plant (Exiqon, Vedbaek, Denmark). The quality and quantity of RNA were evaluated using a NanoDrop spectrophotometer (Thermo Fisher Scientific, Inc., Wilmington, NC, USA). Afterward, cDNA was synthesized by RevertAid RT cDNA Synthesis Kit (Thermo Fisher Scientific). For the synthesis of miR-494 cDNA, a poly (A) tail was added to the 3′-end of the miRNA transcripts by *E. coli *Poly (A) Polymerase (PAP) (New England Biolabs, UK). A primer containing complementary sequence for the poly (A) tail and an adapter sequence were used for reverse transcription reaction. Real-time PCR was done using a SYBR green kit (TaKaRa, Tokyo, Japan) with gene-specific primers according to the manufacturer's protocol. The NAMPT expression level was normalized by GAPDH gene as endogenous control. The expression of miR-494 was normalized by U6 small nuclear RNA. The fold change of NAMPT and miR-494 were calculated by the 2^−ΔΔCT^ method. The primer sequences are listed in (Table 1(Tab. 1)). All reactions were performed in triplicate.

### NAD assay

Total cellular NAD was measured using a colorimetric assay kit (Abcam, London, UK), according to the manufacturer's instructions. Briefly, cells were lysed by lysis buffer and the lysate was deproteinized by perchloric acid to avoid enzymatic digestion. Potassium hydroxide was added to neutralize the acid and balance the pH. Consequently, the lysate and standard solution were reacted with the developer in 96-well plates in triplicate. The plate was incubated at RT in the dark room for 30 minutes following the addition of Cycling enzyme. Finally, the absorbance was measured at 450 nm by a plate reader and the concentration of NAD was determined (Alaee et al., 2017[3]).

### Cell viability assay

Cell viability was measured using water-soluble tetrazolium salt (WST-1) kit (Roche Applied Science, Germany) according to the manufacturer's instructions. The cells were seeded in 96-well plates (1 × 10^4^ cells/well) for 24 h at 37 °C. Then, transfection was performed with miR-494 mimic, inhibitor and appropriate negative controls. Subsequently, 10 μl of WST-1 reagent was added to each well. The plates were mixed gently and incubated for 2-4 h. After the incubation period, the plates were mixed on an orbital shaker for one minute and the absorbance was measured using a microtiter plate reader at 420-480 nm.

### Cell apoptosis

To quantify the effects of miR-494 on cell death, transfected cells were stained with Annexin V-FITC and propidium iodide (PI), using FITC Annexin V apoptosis detection kit (Roche Applied Science, Germany) as suggested by the manufacturer. Briefly, 5×10^5^ cells were seeded in 6-well plates and transfected with miR-494 mimic, inhibitor and appropriate negative controls. Then, the cells were washed twice with cold PBS and incubated with Annexin V-FITC (5 μl) and 10 μl propidium iodide (20 μg/ml) in the dark room for 15 min. Afterwards, apoptosis was analyzed using flow cytometer (FACScan, BD Biosciences, USA) at 488 nm excitation, a bandpass filter at 515 nm for FITC and 600 nm for PI detection, and the data were analyzed by FlowJo 7.6.1 software (Tree Star, Inc). Annexin V-FITC positive cells were reported as apoptotic cells.

### Luciferase assay

The 3′-untranslated region (3^′^-UTR) of NAMPT was amplified by PCR as previously described (Hesari et al., 2018[19]). The digestion products were cloned downstream of the Renilla gene in psiCHECK2 plasmid (Promega, USA), after digestion of the PCR products with XhoI and NotI enzymes (Fermentas, USA). miR-494 mimic (20 pmol) or miR-494 inhibitor (20 pmol) or their negative controls together with the NAMPT-3′-UTR-psiCHECK2 vector were co-transfected into the HEK-293T cells, which were seeded in 12-well plates for 24 h prior to co-transfection. A vector containing the mutant form of the NAMPT miRNA response element (NAMPT-MRE-tandem-mut psiCHECK2) and un-cloned psiCHECK2 plasmid were also co-transfected as negative controls. After 2 days, Renilla luciferase activity was determined using a dual luciferase assay kit (Promega, USA), normalized against firefly luciferase activity. Three independent experiments were performed at different times. 

### Statistical analysis 

All experiments were performed at least three times and data were expressed as means ± standard deviation (S.D.). Statistical analyses were performed by one-way analysis of variance (ANOVA), followed by Tukey's post-hoc test using Graphpad prism version 5.01. For all analyses, *p*-values less than 0.05 were considered to be significant.

## Results

### The basal expression levels of miR-494 and NAMPT in BC and non-tumorigenic human breast cell lines

**Figure 1:**
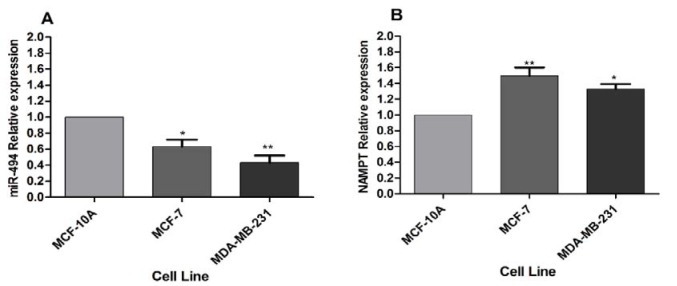
(A) miR-494 expression level and (B) NAMPT relative expression in BC cell lines. MCF-10A was utilized as a control. The results are mean ± SD of at least three independent experiments. * *P* < 0.05; ** *P* < 0.01

The basal expression levels of miR-494 and NAMPT mRNA were measured by qRT-PCR in MCF-7, MDA-MB-231 and MCF-10A cell lines. The results showed that the expression level of miR-494 in MDA-MB-231 and MCF-7 cell lines was significantly lower than that in MCF-10A cells (Figure 1A(Fig. 1)). Conversely, the expression level of NAMPT mRNA in MCF-7 and MDA-MB-231 cells was significantly higher than that in MCF-10A cells** (**Figure 1B(Fig. 1)).

### Overexpression of cellular miR-494 level by miR-494 mimic

**Figure 2:**
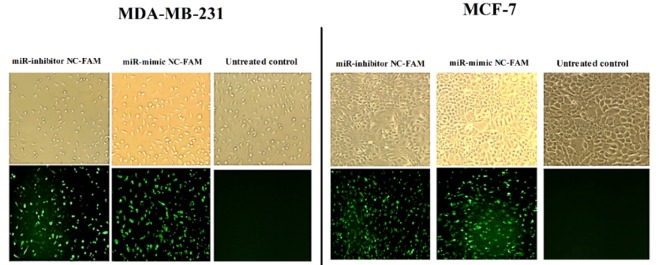
Fluorescence microscopy images of MCF-7 and MDA-MB-231 cells after transfection by FAM-labeled miRNA mimic and inhibitor

**Figure 3:**
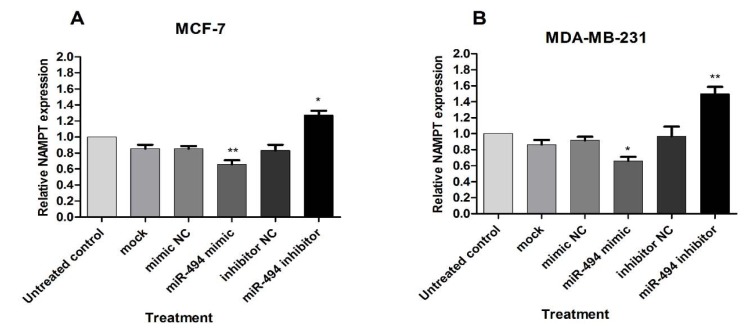
Relative expression of miR-494 in MCF-7 (A) and MDA-MB-231 (B) cell lines after transfection with miR-494 mimic, inhibitor or their negative controls. The results are compared to untreated control and are mean ± SD of three independent experiments. * *P* < 0.05; ** *P* < 0.01; *** *P* < 0.001

To increase and decrease the level of miR-494, BC cells were transfected with miR-494 mimic, miR-494 inhibitor and their negative controls (NC). In order to confirm the transfection, the FAM-labeled miRNA was transfected into the BC cells and observed in the fluorescence microscope. Analysis of the density of emitted fluorescent by the Image J software showed that the transfection was performed with high efficiency (Figure 2(Fig. 2)). Quantification of miR-494 expression level indicated that transfection by miR-494 mimic significantly increased cellular miR-494 level in MDA-MB-231 and MCF-7 cell lines, whereas reduction in the expression level of miR-494 was obtained following transfection with miR-494 inhibitor in these cells **(**Figure 3(Fig. 3)).

### miR-494 regulates NAMPT mRNA and protein expression levels 

**Figure 4:**
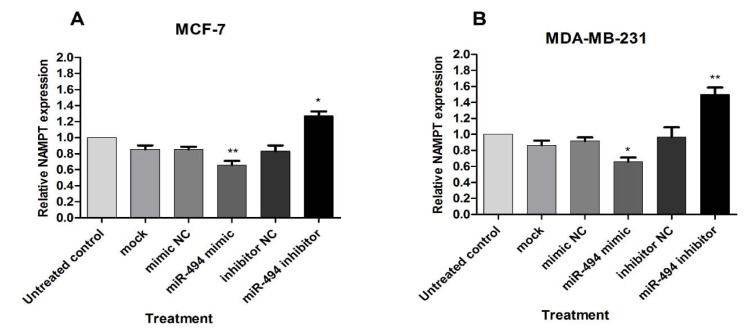
The effect of miR-494 transfection on NAMPT mRNA. The mRNA expression of NAMPT in MCF-7 (A) and MDA-MB-231 (B) cells after transfection with miR-494 mimic, inhibitor or negative controls (NC). * *P* < 0.05; ** *P* < 0.01

The effect of miR-494 on NAMPT mRNA was assessed by qRT-PCR. A significant decrease in NAMPT mRNA was observed in the cells transfected with miR-494 mimic in comparison with control in both MCF-7 and MDA-MB-231 cell lines, while treatment with miR-494 inhibitor significantly increased NAMPT mRNA in these cells (Figure 4A, B(Fig. 4)).

These results showed that miR-494 overexpression inhibited, while miR-494 inhibitor promoted NAMPT levels. It can be suggested that regulation of NAMPT expression level via miR-494 occurs at transcriptional level. 

**Figure 5:**
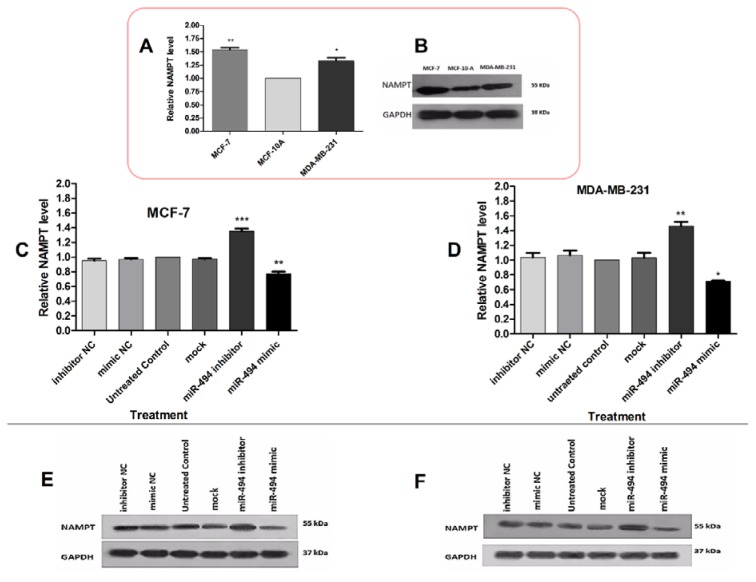
(A) The basal protein levels of NAMPT in MCF-7 and MDA-MB-231 cell lines compared to MCF-10A. (B) A representative Western blotting comparing NAMPT protein level in breast cancer cell lines. The protein levels of NAMPT in MCF-7 (C) and MDA-MB-231 (D) cells after transfection with miR-494 mimic, inhibitor and their negative controls (NC). The results are compared with untreated control group. (E), (F) Representative Western blotting of NAMPT protein after transfection of MCF-7 and MDA-MB-231 cell lines. GAPDH was used as a normalizer. The results are representative of at least three independent experiments. * *P* < 0.05; ** *P* < 0.01; *** *P* < 0.001

The protein level of NAMPT was also assessed in BC cells by Western blotting. The basal protein levels of NAMPT were significantly higher in MCF-7 and MDA-MB-231 cells compared with MCF-10A (Figure 5A, B(Fig. 5)). After transfection, miR-494 mimic significantly decreased NAMPT protein level in both MCF-7 and MDA-MB-231 cells, while miR-494 inhibitor led to an increase in the NAMPT protein level (Figure 5C-F(Fig. 5)).

### Intracellular NAD levels were changed by miR-494 

**Figure 6:**
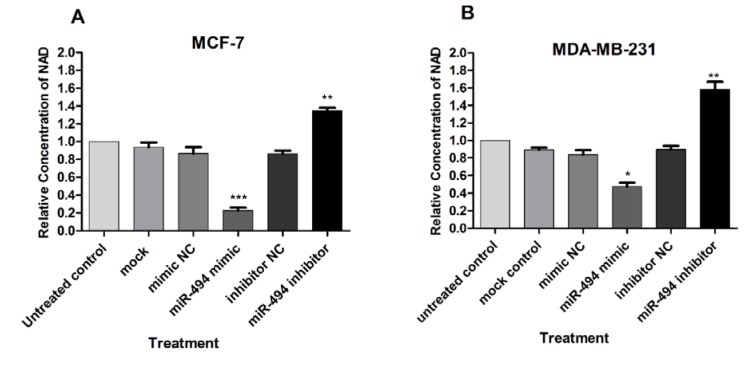
The effect of overexpression of miR-494 on intracellular NAD levels in transfected MCF-7 (A) and MDA-MB-231 (B) cell lines. The results are compared to untreated control and are mean ± SD of three independent experiments. * *P* < 0.05; ** *P* < 0.01; *** *P* < 0.001

NAMPT is a critical enzyme in the NAD biosynthesis pathway. Therefore, it was proposed that inhibition of NAMPT might lead to reduction of NAD content. We examine whether miR-494 is involved in the modification of intracellular NAD levels through modulation of NAMPT expression. As shown in (Figure 6A, B(Fig. 6)), overexpression of miR-494 led to a significant reduction in the intracellular NAD levels in both cell lines, MCF-7 and MDA-MB-231, while in cells transfected with miR-494 inhibitor, elevated level of NAD was observed.

### miR-494 affects cell viability

**Figure 7:**
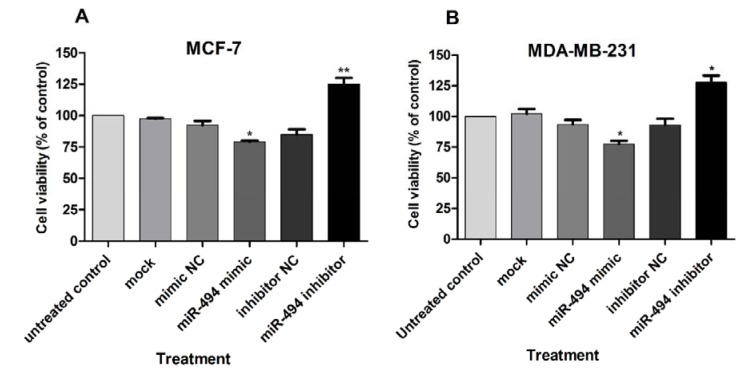
Cell viability (% of control) in MCF-7 (A) and MDA-MB-231 (B) cell lines after transfection with miR-494 mimic, inhibitor or negative controls (NC). The results are compared to untreated control group. The results are mean ± SD of at least three independent experiments. ** *P* < 0.01; * *P* < 0.05

Previous studies have shown that NAMPT and miR-494 are involved in cell viability (Alaee et al., 2017[3], Liu et al., 2015[27], Chen et al., 2015[9], Zhang et al., 2013[54]). Viability of the cells was measured using WST-1. A significant decrease in the viability of both MDA-MB-231 and MCF-7 cells was observed after transfection with miR-494 mimic, compared with controls, while transfection with miR-494 inhibitor significantly increased cell viability (Figure 7A, B(Fig. 7)).

### miR-494 induces cell apoptosis

**Figure 8:**
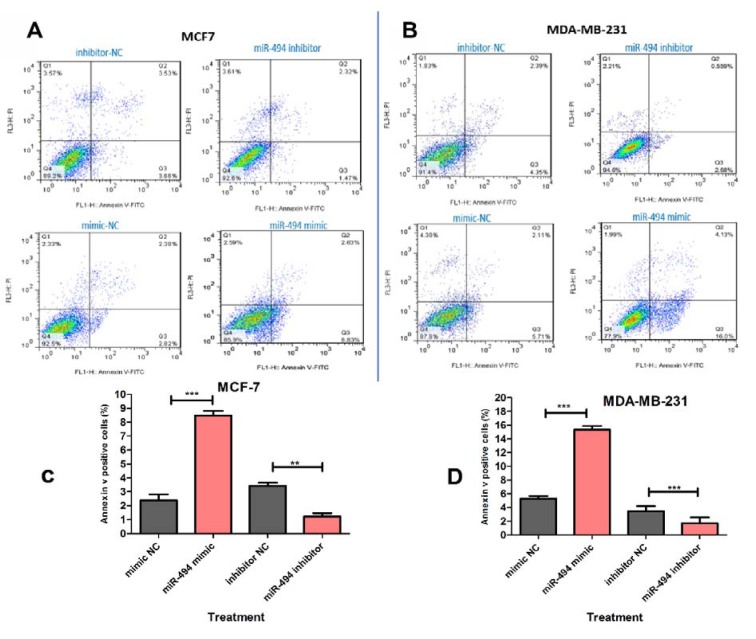
Representative quadrant dot plot of Annexin V-fluorescein isothiocyanate (FITC)/propidium iodide (PI) staining of MCF-7 (A) and MDA-MB-231 (B) cells transfected with miR-494 mimic, inhibitor or negative control (NC). The lower right quadrant shows early apoptotic (annexin V+/PI−). (C, D) Average quantification of apoptotic cells in breast cancer cell lines. The results are compared to negative control group. ** *P* < 0.01; *** *P* < 0.001

As shown in Figure 8A-D(Fig. 8), overexpression of miR-494 significantly induced apoptosis in MCF-7 and MDA-MB-231 cell lines. On the other hand, miR-494 inhibitor could rescue the cells from the endogenous miR-494 and therefore led to a significant decrease in the percentage of early-stage apoptotic cells. 

### miR-494 directly targets NAMPT 3′-UTR

**Figure 9:**
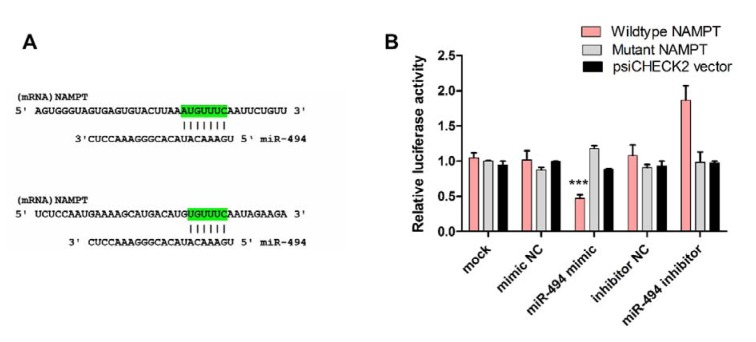
(A) Predicted pairing of miR-494 with the 3'-UTR of NAMPT, using bioinformatics algorithms. (B) The results of luciferase reporter activity assay after transfection of cells with miR-494 mimic, inhibitor or their negative controls (NC) together with the vector containing the wild-type sequence of 3'-UTR of NAMPT (NAMPT-3'UTR Wt). The vector containing the mutated form of the 3'-UTR (NAMPT-3'UTR Mut) and the un-cloned psiCHECK vector were used as negative control. The data are shown as mean ± SD. *** *P* < 0.001

Bioinformatics evaluations showed that miR-494 aligns with two different positions in the 3'-UTR of NAMPT, 977-984 and 1142-1148 (Figure 9A(Fig. 9)). To confirm whether NAMPT is the direct target of miR-494, the relative luciferase activity was determined following co-transfection of constructs containing the 3'-UTR of NAMPT along with miR-494 mimic or inhibitor or their negative controls. The relative luciferase activity after transfection with miR-494 mimic was 0.44±0.05, showing a significant reduction in luciferase activity. miR-494 inhibitor increased the relative luciferase activity to 1.74±0.25 fold to control. The luciferase activity of the cells undergoing co-transfection experiments with miR-494 mimic or inhibitor along with NAMPT-MRE-tandem-mut vector which contained the mutated form of the miR-494 response element was 1.2±0.07 and 1.02±0.16, respectively (Figure 9A-B(Fig. 9)), which were not significantly different from controls.

## Discussion

Breast cancer is a public health challenge with a worldwide growing occurrence that is the main cause of women's death (Siegel et al., 2016[38]; Kartal et al., 2014[24]; Gail, 2015[15]; Yavan et al., 2010[51]). Systemic treatments for BC such as chemotherapy and/or hormonal therapy have many side effects and may sometimes lead to resistance and recurrence. Some molecules have been identified as biomarkers for treatment or diagnosis of BC, however, there is still need for novel therapeutic targets for better management of this disease (Montecucco et al., 2013[29]). 

Studies have shown that NAMPT is significantly increased in BC (Dalamaga et al., 2011[13]) and many other cancers (Reddy et al., 2008[34]). NAMPT, the rate-limiting enzyme of NAD biosynthesis, is considered to play an important role in the cancer cells growth, metastasis and inhibition of apoptosis (Sonoli et al., 2011[41]; Housa et al., 2006[22]; Sheikhpour, 2017[37]). Therefore, targeted inhibition of NAMPT has become an attractive strategy in the management of cancers and other metabolic diseases (Garten et al., 2009[16]). The mechanism by which NAMPT overexpression occurs in cancer is not fully elucidated. miRNAs are currently accepted as important players in post-transcriptional control of gene expression and many aberrations in cancer cells may be attributed to the derangement of these molecules (Slaby et al., 2017[39]). Thus, miRNAs may be responsible for the dysregulation of NAMPT levels in cancer. 

In this study, we investigated the relationship between miR-494 and NAMPT. Our results revealed that the basal expression level of miR-494 in BC cell lines was significantly lower than that in the non-tumorigenic human breast cells (MCF-10A). Consistently, Song et al. (2015[40]) and Zhan et al. (2017[53]) reported that miR-494 expression was down-regulated in BC cells. We also found that the basal protein levels of NAMPT in MCF-7 and MDA-MB-231 cells were significantly higher than MCF-10A, which is consistent with findings from previous studies (Lee et al., 2011[26]; Zhu et al., 2016[55]; Sharif et al., 2016[36]). We showed that there is an inverse relationship between expression of miR-494 and NAMPT in MCF-7 and MDA-MB-231 cell lines. To up-regulate miR-494 in BC cells, we transfected the cells with miR-494 mimic and to reduce the endogenous miR-494, its inhibitor was used. Our results demonstrated that overexpression of miR-494 in MCF-7 and MDA-MB-231 cells significantly decreased the levels of NAMPT mRNA and protein. These results revealed that miR-494 controls NAMPT expression at the post-transcriptional level and that the reduction of NAMPT mRNA by miR-494 causes the decline in NAMPT protein expression. 

Some studies have shown the effect of different miRNAs on NAMPT expression. Zhang et al. (2013[54]) showed that miR-26b inhibited NAMPT expression at the protein and mRNA levels by binding to the its 3′-UTR in colorectal cancer cell lines (SW480, SW1116, LoVo, and HCT116). Hesari et al. (2018[19]) reported that miR-206 inhibited NAMPT expression at the translational level by directly binding to its 3′-UTR. Adyshev et al. also showed that miR-374a and miR-568 reduced the level of mRNA and protein of NAMPT in human lung endothelial cells (Adyshev et al., 2014[1]). 

To confirm that miR-494 directly interacts with NAMPT mRNA, a dual-luciferase reporter assay was performed. miR-494 mimic decreased the luciferase activity while miR-494 inhibitor augmented the luciferase activity. These results indicate that NAMPT down-regulation is caused by the direct binding of miR-494 to the 3'-UTR of NAMPT mRNA and it is not an off-target effect. 

Some targets other than NAMPT have also been introduced for miR-494. Liu et al. have recently demonstrated that miR-494 negatively regulates c-Myc and SIRT1 expression directly by binding to the 3'-UTR of c-Myc and SIRT1 and in this fashion it could block the positive loop of c-Myc and SIRT1 (Liu et al., 2015[27]). Thus miR-494 not only inhibits SIRT1 but also reduces NAD as its main substrate by targeting NAMPT. 

NAD is generated by NAMPT-mediated conversion of NAM to NMN, which finally gives rise to NAD by nicotinamide/nicotinic acid mononucleotide adenylyltransferase (NMNAT) (Houtkooper et al., 2010[23], Revollo et al., 2007[35]). Evaluation of cells treated with NAMPT inhibitors *in vitro* has revealed a rapid decrease in intracellular NAD (Watson et al., 2009[47], Xiao et al., 2013[49]). Our data showed that NAMPT inhibition in response to overexpression of miR-494 caused a significant decline in the NAD levels while miR-494 inhibitor increased cellular NAD level, indicating that miR-494 can be considered an effective regulator of cellular NAD status. 

Taking into account the various functions of NAD, the effects of NAMPT inhibition on cellular physiology are profound. It has been shown that inhibition of NAMPT leads to an induction of apoptosis and reduction of cell survival in cancer cell lines concomitant with the decline in NAD levels (Alaee et al., 2017[3]; Hasmann and Schemainda, 2003[18]; Yang et al., 2007[50]; Holen et al., 2008[21]) . Here we revealed that up-regulation of miR-494 reduced cell viability and induced apoptosis in BC cells. Consistently, Liu et al. have reported that overexpression of miR-494 mimic can reduce cell viability in pancreatic cancer cells (Liu et al., 2015[27]). In the current study we also showed that miR-494 inhibitor prevented apoptosis and augmented cell viability by rescuing the cells from the effects of cellular endogenous miR-494, pointing out that the reduction of miR-494 might lead to improved cell survival. Apoptosis plays a crucial role in cancer management and therefore is considered as a protective mechanism against tumor progression and metastasis (Pfeffer and Singh, 2018[33]; Hickman, 1992[20]). Moreover, the anticancer activity of most of the chemotherapeutic drugs relies on inducing apoptosis. As a result, inhibition of apoptosis is considered a major factor in resistance to therapy and limits the effectiveness of anticancer treatments. Thus down-regulation of miR-494 can have deleterious effects on BC, while its upregulation is beneficial for BC management. Our findings demonstrated that miR-494 is down-regulated in BC cells, and is able to reduce NAD level by direct targeting of NAMPT and consequently decrease cell viability and induce apoptosis. These findings suggested that miR-494 may serve as a potential candidate in human BC therapeutics. More *in vitro *and* in vivo* studies are essential to further elucidate the functions of miRNA as a tumor suppressor.

## Acknowledgements

The authors are thankful to the staff of the Biochemistry Departments of Iran University of Medical Sciences (IUMS). This study was financially supported by a grant from Iran University of Medical Sciences (Number: 94-02-30-26131).

## Conflict of interest

The authors declare that they have no conflicts of interest.

## Figures and Tables

**Table 1:**
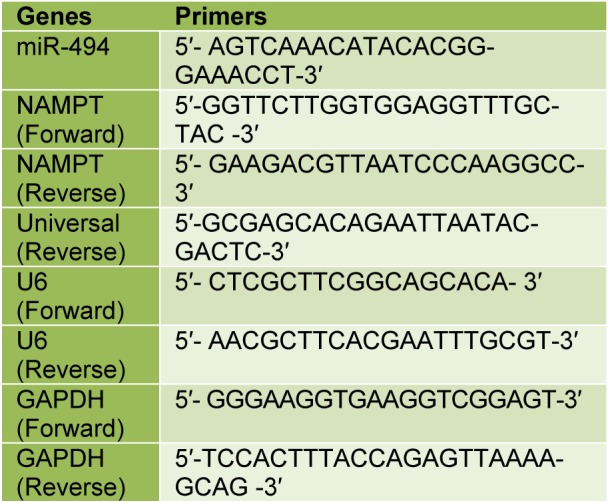
Sequence of primers
